# Geographic Association Between Income Inequality and Obesity Among Adults in New York State

**DOI:** 10.5888/pcd15.180217

**Published:** 2018-10-11

**Authors:** Daniel Kim, Fusheng Wang, Chrisa Arcan

**Affiliations:** 1 Department of Biomedical Informatics and Department of Computer Science, Stony Brook University, Stony Brook, New York; 2 Department of Family, Population, and Preventive Medicine, Stony Brook University, Stony Brook, New York

## Abstract

**Introduction:**

In addition to economic factors and geographic area poverty, area income inequality — the extent to which income is distributed in an uneven manner across a population — has been found to influence health outcomes and obesity. We used a spatial-based approach to describe interactions between neighboring areas with the objective of generating new insights into the relationships between county-level income inequality, poverty, and obesity prevalence across New York State (NYS).

**Methods:**

We used data from the 2015 American Community Survey and 2013 obesity estimates from the Centers for Disease Control and Prevention for NYS to examine correlations between county-level economic factors and obesity. Spatial mapping and analysis were conducted with ArcMap. Ordinary least squares modeling with adjusting variables was used to examine associations between county-level obesity percentages and county-level income inequality (Gini index). Univariate spatial analysis was conducted between obesity and Gini index, and globally weighted regression and Hot Spot Analysis were used to view spatial clustering.

**Results:**

Although higher income inequality was associated with lower obesity rates, a higher percentage of poverty was associated with higher obesity rates. A higher percentage of Hispanic population was associated with lower obesity rates. When tested spatially, higher income inequality was associated with a greater decrease in obesity in southern and eastern NYS counties than in the northern and western counties, with some differences by sex present in this association.

**Conclusion:**

Increased income inequality and lower poverty percentage were significantly linked to lower obesity rates across NYS counties for men. Income inequality influence differed by geographic location. These findings indicate that in areas with high income inequality, currently unknown aspects of the environment may benefit low-income residents. Future studies should also include environmental factors possibly linked to obesity.

## Introduction

Economic factors have been linked to numerous health outcomes, including obesity (1). However, research on area income inequality — the extent to which income is distributed unevenly across a population — and obesity rates is limited and inconsistent, because income inequality is a contextual variable specific to geographic scale and is differentially associated with social conditions. The relationship between income inequality and obesity changes by geographic area and is not fully understood.

In the United States, obesity is related to poverty, low individual income, and food-insecurity (1). A study that used data from the 2003–2008 National Health and Nutrition Examination Survey showed that at the tract and county levels, high degrees of income inequality was correlated with low obesity rates (2), suggesting that community affluence has a positive effect on residents’ lifestyles. Similarly, city-level and tract-level income inequality was negatively associated with body weight in Los Angeles county in 2000–2001 (3). A study using the Behavioral Risk Factor Surveillance System (BRFSS) found that a high prevalence of income inequality was associated with reduced odds of obesity among non-Hispanic white women (4). To our knowledge, previous studies have not used spatial regression methods to examine the relationship between area income inequality and obesity rates. To address this research gap, we used spatial analysis to examine associations between small-area income inequality and obesity among adults in New York State (NYS). We hypothesized that income inequality would have an inverse relationship with obesity rates and that a geographic difference exists between the two.

## Methods

### Data sources

Our study used a cross-sectional design of publicly available data sources to create estimates related to NYS residents. Data from the American Community Survey (ACS) (https://www.census.gov/programs-surveys/acs) were used for all independent variables, including area poverty prevalence and area income inequality. ACS is an annual survey conducted by the US Census Bureau throughout the United States and provides annual estimates of a series of monthly samples of people living in housing units, such as houses or apartments, and in institutional and noninstitutional group quarters, such as correctional facilities, mental hospitals, college dormitories, military barracks, and shelters. The Census Bureau uses several data collection methods (internet, mailed paper questionnaire, telephone, personal visit) to ensure representation of the US population. The ACS survey is mandatory by law, resulting in an extremely high response rate. Participants were excluded for refusal to participate based on legal or other reasons, insufficient data, inability to locate participants, temporary absences from their place of residence, and language barriers. ACS is conducted in English, meaning that results cannot be retrieved if interpreters are unavailable. Our study used ACS 5-year estimates (2011–2015), representing 790,051 observations. Even at a 99.5% confidence interval, the necessary sample size to ensure correct estimates for the NYS population was 38,341, less than the number of participants in the ACS survey.

County-level income inequality was measured by the Gini coefficient, or Gini index, which represents income dispersion across an area, assigning values from 0 to 1: the higher the number, the greater an area’s income inequality. The numerator of the coefficient is the area between the Lorenz curve of the distribution and the uniform distribution line; the denominator is the area under the uniform distribution line. We converted this ratio into an index by multiplying each value by 100. Gini index was the only variable not separated by sex. In the ACS data set, racial groups were recorded as counts and were converted to percentages by dividing the counts for each racial group by the total estimated number of people in each county. We used the Gini index in this study because it is the most commonly used measure of income inequality; however, we acknowledge the existence of other measures, such as Atkinson’s measures, Theil’s T, and Theil’s L, and that our results may not necessarily have held if these other measures were used instead of the Gini index (5,6).

The dependent variable, obesity prevalence, was drawn from the Centers for Disease Control and Prevention (CDC) statistical estimates (7,8). These were based on the Census Bureau’s Population Estimates Program and the 2013 BRFSS (9), which was conducted via telephone interview. However, these estimates also include statistical adjustments designed to reduce the random sampling’s inherent randomness (7). Obesity was defined as a body mass index (BMI, kg/m^2^) of 30 or greater and was measured by physical examinations at the county level.

### Statistical methods

We examined the association between county-level independent variables and obesity prevalence with ArcMap (Esri) by using ordinary least squares (OLS). OLS is a variation of linear regression, a statistical method that examines associations between multiple independent variables and a single dependent variable; once the assumptions are satisfied, the regression output indicates the strength of the association between the dependent variable and each of the independent variables. These assumptions, include linear parameters, random sampling, no multicollinearity, no autocorrelation, a conditional mean of zero, and normally distributed error terms; all of them were satisfied, meaning that our OLS models are efficient and represent a linear unbiased estimator of variable coefficients.

Final models included county-level Gini index, poverty percentage (defined as having an income below the Federal Poverty Level), adjusted for median age, percentage African-American, percentage Hispanic, percentage married, and percentage with at least a high school education. Statistical significance was set at *P* < .05. Interactions between the sex ratio with each of the other independent variables were tested. Because we found significant interactions between sex and the Gini index, analyses were conducted separately by sex. After these analyses, we found that coefficients and *P* values did not differ by sex; therefore we performed the analysis with both sexes combined.

Two spatial tests, geographically weighted regression (GWR) and Getis-Ord GI* Hot Spot Analysis (Esri), were used to add a different dimension to our analysis. GWR created a separate ordinary least squares (OLS) model for every county while considering spatial factors, such as the distances and OLS models of neighboring counties. GWR measured relationships that vary across space, whereas OLS linear regression assumes these relationships apply equally over an entire geographic area (9). We performed univariate GWR with Gini index as our independent variable, with both Gini index and obesity prevalence first matched to counties in a NYS ArcMap shapefile.

Hot Spot Analysis was conducted on the GWR regression results; this test determines whether the different coefficients of the Gini index variable for each county that GWR returned are randomly dispersed, or whether unusually high or unusually low values are clustered together. Hot Spot Analysis tests for clusters of similar values in a set of spatial data, indicating when similar values are close to one another. The method is specific, enabling us to detect possible local spatial associations whereas other methods, such as Moran’s I, does not (10).

Although standard OLS regression makes one model for the entire state, giving an overall sense of a variable’s effect on obesity rates, GWR combined with Hot Spot Analysis provides information about the degree of effect a variable has in different areas. This allowed for observation of differences in the effect of income inequality on obesity prevalence across NYS.

## Results

The median age in our data set of the NYS population was 38.1 years; 48.5% were men, 15.6% were black, 18.4% were Hispanic, 44.5% were married, and 85.6% were high school graduates. During the time that these data were collected, the response rate varied by county; however, for NYS, the overall response rate of housing units was 93.3%, and the overall response rate of group quarters was 95.2%.

The OLS regression showed that among all adults, a higher county-level Gini index (or higher inequality) (β, −0.37; *P* = .01) and a higher percentage of Hispanic population (β, −0.22; *P* = .009) was significantly associated with a lower obesity rate. In contrast, a higher percentage of county-level poverty (β, 0.42; *P* = .004) and higher percentage of being married (β, 0.22; *P* = .03) was associated with a higher obesity rate (Table 1). Then in separate analyses, the same significant associations were observed among men and women with the exception of marital status, which was significant among men (Table 2) but not among women (Table 3). We used Hot Spot Analysis to test for spatial autocorrelation, and none was found. Variance inflation factor values of all variables were measured, with none exceeding 5, a benchmark for moderate multicollinearity.

**Table 1:** Effects of Income Inequality^a^, Poverty Percentage, and Sociodemographic Variables on Obesity at the County Level Among Adults in New York State^b^

Variable	β Coefficient	Standard Error	*P* Value^c^
Intercept^d^	16.91	21.06	.43
Gini index	−.37	.14	.01
Poverty^e^, %	.42	.14	.004
Median age	.09	.10	.36
African-American, %	.14	.10	.14
Hispanic, %	−.22	.09	.009
Married, %	.22	.10	.03
High school graduate, %	.08	.16	.64

a Calculated by Gini index drawn from 5-year estimates of the American Community Survey for 2015.

b Based on an ordinary least squares multivariable linear regression model. Poverty percentage and sociodemographic variables were drawn from 5-year estimates of the American Community Survey for 2015. The dependent variable, obesity percentage, is based on 2013 CDC County Data Indicators (https://www.cdc.gov/diabetes/data/countydata/countydataindicators.html) estimates based on the BRFSS (Behavioral Risk Factor Surveillance System) survey (9).

c *P* values were calculated by using the ordinary least squares statistical test. Significance was set at *P* < .05.

d The intercept of the OLS regression model. Defined, in this case, as the expected value of obesity prevalence if all independent variables used in the equation are set to 0.

e Defined as percentage of population with annual incomes below the Federal Poverty Level.

**Table 2:** Effects of Income Inequality^a^, Poverty Percentage, and Sociodemographic Variables on Obesity at the County Level Among Adult Men in New York State^b^

Variable	β Coefficient	Standard Error	*P* Value^c^
Intercept^d^	35.68	15.89	.03
Gini index	−.41	.13	.004
Poverty^e^, %	.31	.14	.03
Median age	.04	.10	.68
African-American, %	.07	.09	.48
Hispanic, %	−.26	.08	<.001
Married, %	.21	.08	.01
High school graduate, %	−.04	.13	.76

a Calculated by Gini index drawn from 5-year estimates of the American Community Survey for 2015.

b Based on an ordinary least squares multivariable linear regression model. Poverty percentage and sociodemographic variables were drawn from 5-year estimates of the American Community Survey for 2015. The dependent variable, obesity percentage, is based on 2013 CDC estimates based on the BRFSS (Behavioral Risk Factor Surveillance System) survey (9).

c *P* values were calculated by using the ordinary least squares statistical test. Significance was set at *P *< .05.

d The intercept of the OLS regression model. Defined, in this case, as the expected value of obesity prevalence if all independent variables used in the equation are set to 0.

e Defined as percentage of population with annual incomes below the Federal Poverty Level.

**Table 3:** Effects of Income Inequality^a^, Poverty Percentage, and Sociodemographic Variables on Obesity at the County Level Among Adult Women in New York State^b^

Variable	β Coefficient	Standard Error	*P* Value^c^
Intercept^d^	19.82	22.92	.39
Gini index	−.34	.15	.03
Poverty, %^e^	.38	.13	.004
Median age	.08	.10	.40
African-American, %	.18	.10	.07
Hispanic, %	−.20	.09	.03
Married, %	.15	.10	.14
High school graduate, %	.05	.18	.80

a Calculated by Gini index drawn from 5-year estimates of the American Community Survey for 2015.

b Based on an ordinary least squares multivariable linear regression model. Poverty percentage and sociodemographic variables were drawn from 5-year estimates of the American Community Survey for 2015. The dependent variable, obesity percentage, is based on 2013 CDC estimates based on the BRFSS (Behavioral Risk Factor Surveillance System) survey (9).

c *P* values were calculated by using the ordinary least squares statistical test. Significance was set at *P* < .05.

d The intercept of the OLS regression model. Defined, in this case, as the expected value of obesity prevalence if all independent variables used in the equation are set to 0.

e Defined as percentage of population with annual incomes below the Federal Poverty Level.

**Figure 1:**
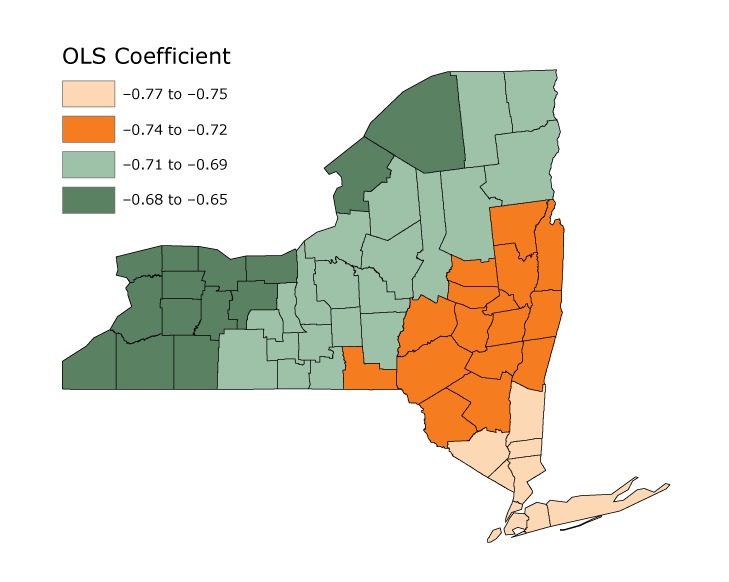
Results of geographically weighted regression (GWR) tests for men, mapping the individual ordinary least squares (OLS) coefficient constructed by GWR to each county in New York State. Data are from the American Community Survey and from CDC County Data Indicators estimates (11).

**Figure 2:**
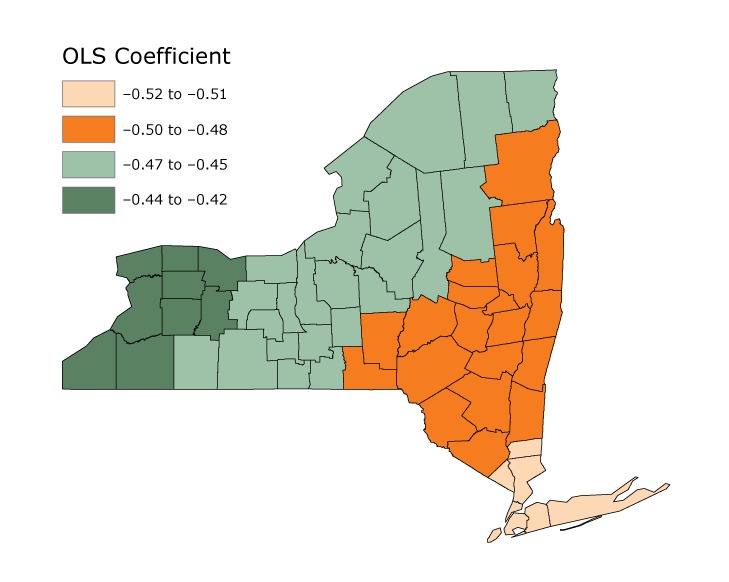
Results of geographically weighted regression (GWR) tests for women, mapping the individual ordinary least squares coefficient constructed by GWR to each county in New York State. Data are from the American Community Survey and from CDC County Data Indicators (11).

The GWR analysis showed that a 1% increase in income inequality was associated with a greater decrease in obesity prevalence in southern NYS than in the western state for both sexes. The effect of the Gini index on obesity prevalence was highest in southern and eastern NYS, but showed a downward trend toward the north and west. These associations were stronger among men (Figure 1) than among women (Figure 2), just as the OLS models predicted.

Hot Spot Analysis tests confirmed GWR results: a large area exists in the southeast where the effect of the Gini index is unusually high compared with its surrounding areas, and a large area in the west where this effect is unusually low compared with neighboring areas. From the results of the GWR and Hotspot tests, we observed a connection between the differing effects of income inequality (Gini index) and its relation to geographical direction in NYS. Moving east the absolute effect of income inequality on obesity increased, whereas moving west it, decreased, which the Hot Spot test confirmed.

## Discussion

Our study examined associations between obesity prevalence and county-level income inequality and poverty percentage among adults in NYS. As we hypothesized, income inequality was inversely associated with obesity prevalence, and a difference in the geographical effect on income inequality and obesity was observed. Our findings using spatial analyses can help public health officials and lawmakers to tailor health initiatives to different geographical areas, thereby improving the sustainability of these initiatives on the well-being of the population.

The negative correlation of income inequality with obesity is not unilateral; a study of 21 developed countries showed that income inequality was positively correlated with obesity prevalence in men and women (12). Social inequalities were found to have a greater effect on obesity in women in a study of 11 member countries of the Organization for Economic Cooperation and Development (OECD), which include the United States (13). Our study found that income inequality had a greater effect on obesity among men than among women. These conflicting findings may be due to the use of different types of measurements, the inclusion of different countries in the studies, and the geographic area studied, such as NYS. The area level studied was shown to have differing effects of income inequality on other health outcomes (14).

Country-level studies examining national data suggested a detrimental effect of high income inequality to mean BMI and prevalence of obesity (15). A study of 68 countries noted that obesity prevalence was greater among women than among men in countries with a high Gini index (16). Another study using national data from the Behavioral Risk Factor Surveillance System found little to no association between income inequality and obesity in race–sex stratified groups in metropolitan areas (4). Similarly, using national data from Spain’s 2001 National Health Survey, a study found no association between income inequality and BMI (17). A multinational study associated high income inequality at the national level with increases in obesity prevalence; this association disappeared when the United States and Mexico were excluded from their model (18). In contrast, a study using county and tract data found an association between income inequality and BMI similar to our findings, leading us to think that differences in the overall geographical area measured may contribute to differences in the associations between income inequality and obesity.

When considering poverty, our study agrees with similar studies conducted among populations of adult men and women in various countries. A study of Canadian men and women found that rich men and poor women were more likely to be obese (19). Although that study did not measure individual income, poverty percentage was positively associated with obesity among women. Low area socioeconomic status, low-cost food stores, low education attainment, and individual income have been associated with high obesity rates in adults living in Seattle, Washington, and Paris, France (20). In England, a study of adults aged 18 to 75 showed that social and economic gradients existed for obesity in both sexes, with lower socioeconomic status associated with higher rates of obesity, and that this trend had not changed significantly in more than a decade (21).

A study that examined Gini index in adults at the US county and tract levels showed that the addition of potential confounders changed the degree of the association between income inequality and obesity, because area level factors such as neighborhood environment (eg, availability of parks and recreation, healthy food), and local policies may have an effect on residents’ weight status (2). One study of US counties showed that geographical differences in obesity rates can be explained through physical activity and food environments, along with settlement patterns and transportation habits (22). However, this may be due to other factors; income inequality has been associated with low rates of physical activity, which may contribute in part to our findings (23). Future studies may test these correlations by including potential factors as mediators, especially in an area-based study that takes into account context factors, such as distance from parks or other neighborhood services or conditions (23).

County-level poverty was positively associated with obesity in our study. A study of 1,150 children that used data from the National Institute of Child Health and Human Development Study of Early Child Care and Youth Development found that poverty in very early life was associated with obesity in adolescence (24). Some studies differentiated socioeconomic differences by sex, such as one that used data from the 2001–2009 Korea National Health and Nutrition Examination Survey to study Korean adults (25). That study found that lower education was associated with higher obesity rates in women, and higher income was related to higher obesity rates in men. Another study that looked at several US counties found a positive relationship between poverty and obesity (1), suggesting that the positive relationship could have been due to lower physical activity rates of people living in poor counties, which introduces another possible variable in the relationship between county-level poverty and obesity rates.

Studies looking at the relationship between poverty and obesity, have used the term “poverty-obesity paradox” to indicate the positive relationship often found between poverty and obesity. Similar results were observed among the elderly by using data from the Survey of Health, Ageing, and Retirement and from the English Longitudinal Study of Ageing (26). Another study indicated a relationship between food insecurity and obesity through resource scarcity, suggesting that obesity is a response to a threatened food supply (27).

Our study has numerous strengths, including the use of OLS regression and the relatively high number of counties that NYS has compared with other states. The data used were CDC estimates derived from statistical estimates that sought to minimize error, and from ACS data, which is a conglomerate of half a decade of data collected from a high number of interviews. Another strength of our study is the use of GWR and Hot Spot Analysis to determine obesity prevalence geographically, a combined approach that has not often been tried in the literature, allowing for spatial analysis. These results are also highly generalizable. This study was conducted with large data sets, improving the generalizability of the findings. A similar approach can be conducted for the entire United States as needed.

Our study also had limitations. The study’s cross-sectional design limited our ability to infer causality. Also, some of the variables in the BRFSS dataset are self-reported and may be subject to desirability or recall bias (28).

In conclusion, we found that income inequality was inversely associated with obesity prevalence in NYS counties, although this effect differed by sex. Also, the effect of income inequality differed geographically; income inequality was weaker in western NYS and stronger in the east. This trend did not differ by sex. Poverty percentage, however, was positively associated with obesity. Future studies can use spatial-based multiple regression models by introducing potential area-level factors that may contribute to the differing geographical effects of income inequality on obesity. The findings can help design effective programs that will be tailored to address the unique needs of the geographic locations, thus improving the sustainability of health outcomes.
